# Surgical approaches to intramedullary spinal cord astrocytomas in the age of genomics

**DOI:** 10.3389/fonc.2022.982089

**Published:** 2022-09-06

**Authors:** Andrew M. Hersh, George I. Jallo, Nir Shimony

**Affiliations:** ^1^ Department of Neurosurgery, Johns Hopkins University School of Medicine, Baltimore, MD, United States; ^2^ Department of Neurosurgery, Johns Hopkins Medicine, Institute for Brain Protection Sciences, Johns Hopkins All Children’s Hospital, St. Petersburg, FL, United States; ^3^ Department of Surgery, St. Jude Children’s Research Hospital, Memphis, TN, United States; ^4^ Le Bonheur Neuroscience Institute, Le Bonheur Children’s Hospital, Memphis, TN, United States; ^5^ Department of Neurosurgery, University of Tennessee Health Science Center, Memphis, TN, United States

**Keywords:** intramedullary, astrocytoma, tumor, spinal cord, resection, biomarkers, targeted therapy, genetic

## Abstract

Intramedullary astrocytomas represent approximately 30%–40% of all intramedullary tumors and are the most common intramedullary tumor in children. Surgical resection is considered the mainstay of treatment in symptomatic patients with neurological deficits. Gross total resection (GTR) can be difficult to achieve as astrocytomas frequently present as diffuse lesions that infiltrate the cord. Therefore, GTR carries a substantial risk of new post-operative deficits. Consequently, subtotal resection and biopsy are often the only surgical options attempted. A midline or paramedian sulcal myelotomy is frequently used for surgical resection, although a dorsal root entry zone myelotomy can be used for lateral tumors. Intra-operative neuromonitoring using D-wave integrity, somatosensory, and motor evoked potentials is critical to facilitating a safe resection. Adjuvant radiation and chemotherapy, such as temozolomide, are often administered for high-grade recurrent or progressive lesions; however, consensus is lacking on their efficacy. Biopsied tumors can be analyzed for molecular markers that inform clinicians about the tumor’s prognosis and response to conventional as well as targeted therapeutic treatments. Stratification of intramedullary tumors is increasingly based on molecular features and mutational status. The landscape of genetic and epigenetic mutations in intramedullary astrocytomas is not equivalent to their intracranial counterparts, with important difference in frequency and type of mutations. Therefore, dedicated attention is needed to cohorts of patients with intramedullary tumors. Targeted therapeutic agents can be designed and administered to patients based on their mutational status, which may be used in coordination with traditional surgical resection to improve overall survival and functional status.

## Introduction

Astrocytomas of the spinal cord are rare tumors accounting for nearly 30%–40% of all intramedullary spinal cord tumors (1, 2). They constitute the most common type of intramedullary tumor in children, representing 40%–60% of intramedullary spinal cord neoplasms (1, 3, 4). Intramedullary astrocytomas are frequently classified according to the World Health Organization (WHO) categorization system using a 1 through 4 grading system. Traditionally, these grades corresponded to their histological features, and included pilocytic astrocytomas as Grade 1, diffuse or fibrillary tumors as Grade 2, anaplastic astrocytomas as Grade 3, and glioblastoma (GBM) as Grade 4 (5, 6). More recently, updated guidelines incorporate genetic markers into the grading system, in addition to traditional histological features (7). Low-grade tumors (Grades 1 and 2) account for most intramedullary astrocytomas, whereas high-grade lesions (Grades 3 and 4) represent about 25% of cases and confer a poor prognosis (5). Most pediatric tumors are low-grade pilocytic astrocytomas, whereas high-grade lesions occur more frequently in adults (1, 8–10).

Astrocytomas of the spinal cord tend to present eccentric to the central canal and frequently present with poor margins (5, 11). Infiltrative tumors tend to be higher grade, whereas low-grade tumors tend to be more circumscribed, particularly pilocytic astrocytomas (12). Novel techniques in image analysis and radiomics are increasingly being used to classify the pathological diagnosis prior to resection. As one example, a lateralization index has been developed to consider the distance between the center of the cord and the lateral edge of the tumor, with astrocytomas displaying greater dispersion than ependymomas (13). Machine learning techniques can be applied to classify the tumor’s malignant status or automatically segment the spinal cord to quantify the tumor morphology (14, 15). Indeed, the tumor molecular landscape can affect its appearance on imaging in ways that are perceptible only by machine learning and high-throughput radiomic algorithms. This new field of radiogenomics uses quantitative imaging techniques to relate imaging features with the genotype of the tumor, allowing prediction of prognosis, stratification of patients, and determination of therapy (16).

**Figure 1:**
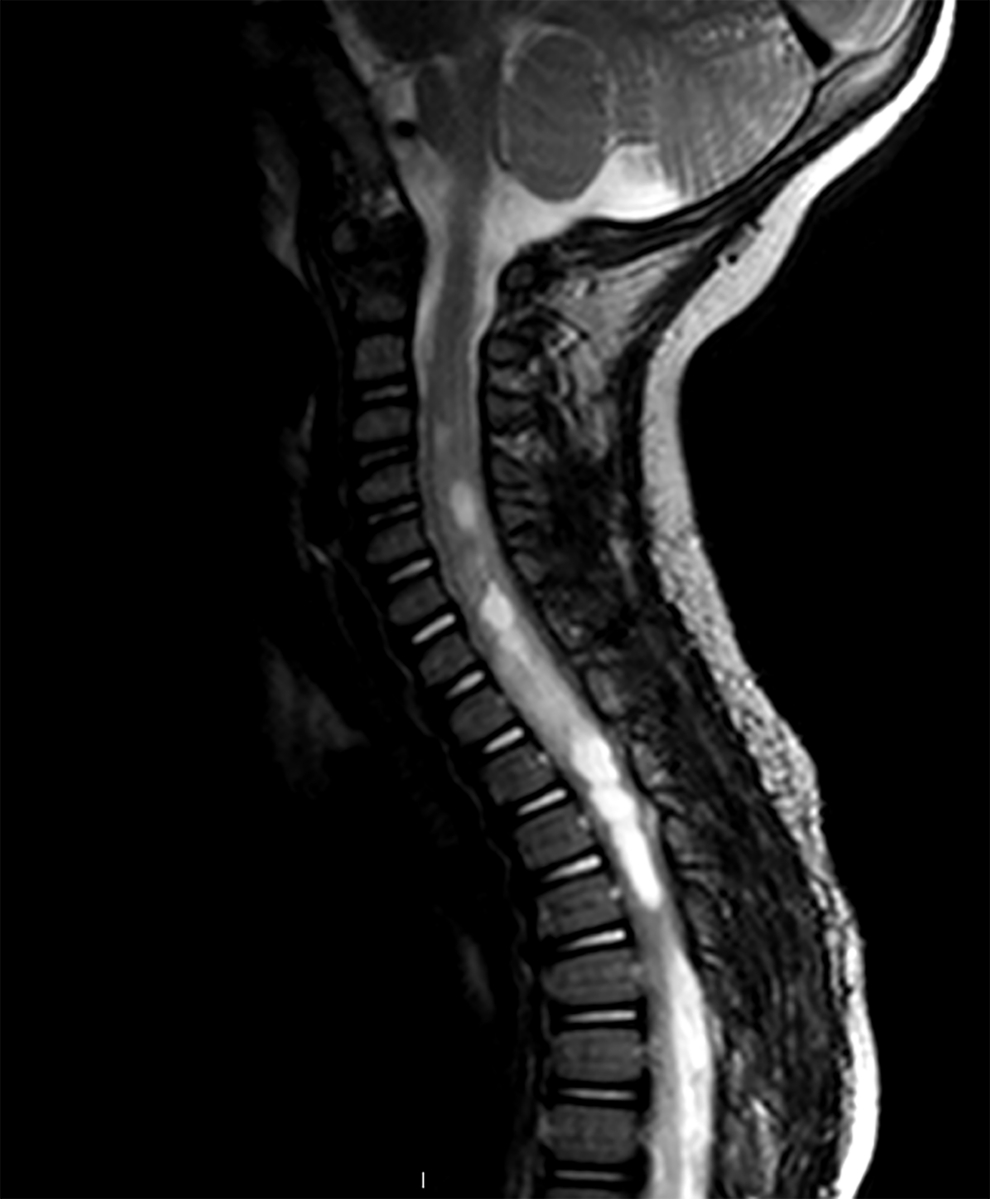
Sagittal T2-weighted MRI of pediatric patient with an intramedullary mass extending from C4-T3. Increased T2 signal is seen both cranially and caudally to the tumor. The lesion appears enhancing, although several areas of central non-enhancement consistent with necrosis are visible. Pathology was consistent with a Grade 2 astrocytoma.

Intramedullary tumors can present with nonspecific symptoms, such as axial back pain and radiculopathy, complicating diagnosis (10). Growth of the neoplasm results in progressive deformation of the spinal cord, contributing to numbness and sensory deficits, weakness and motor deficits, and bowel and bladder dysfunction (17, 18). Astrocytomas typically present hypo-or isointense on T1-weighted and hyperintense on T2-weighted magnetic resonance imaging (MRI) and can include syrinx formation (18) (**Figure 1**).

Survival for patients with intramedullary astrocytomas is poorer compared to ependymomas and hemangioblastomas, with reported overall rates ranging from 40%–65% over 15 years (17). These poorer outcomes reflect the infiltrative nature of astrocytomas, resulting in low rates of gross total resection (GTR) or near total resection. In addition, their poorly delineated margins can result in increased post-operative morbidity that contributes to reduced survival (18). The WHO grade is an important prognostic factor, as high-grade astrocytomas can progress aggressively in short timespans, often conferring mortality within a few months (3, 19–21). Older age has also been identified as a risk factor of increased mortality (3). Higher-grade astrocytomas are also less likely to respond to adjuvant therapy. Nonetheless, surgical treatment with adjuvant therapy is often advocated for intramedullary astrocytomas. However, this view does not account for genetic phenotypes that influence survival or response to therapy, and new insights into the molecular markers of astrocytomas may challenge traditional approaches (22). Although there is an overlap in the genetic profile of intracranial and intramedullary astrocytomas, distinct differences have been noted that these can influence prognosis and treatment, and intramedullary astrocytomas should therefore be analyzed separately from their intracranial counterpart (23).

## Management

### Surgery

**Figure 2:**
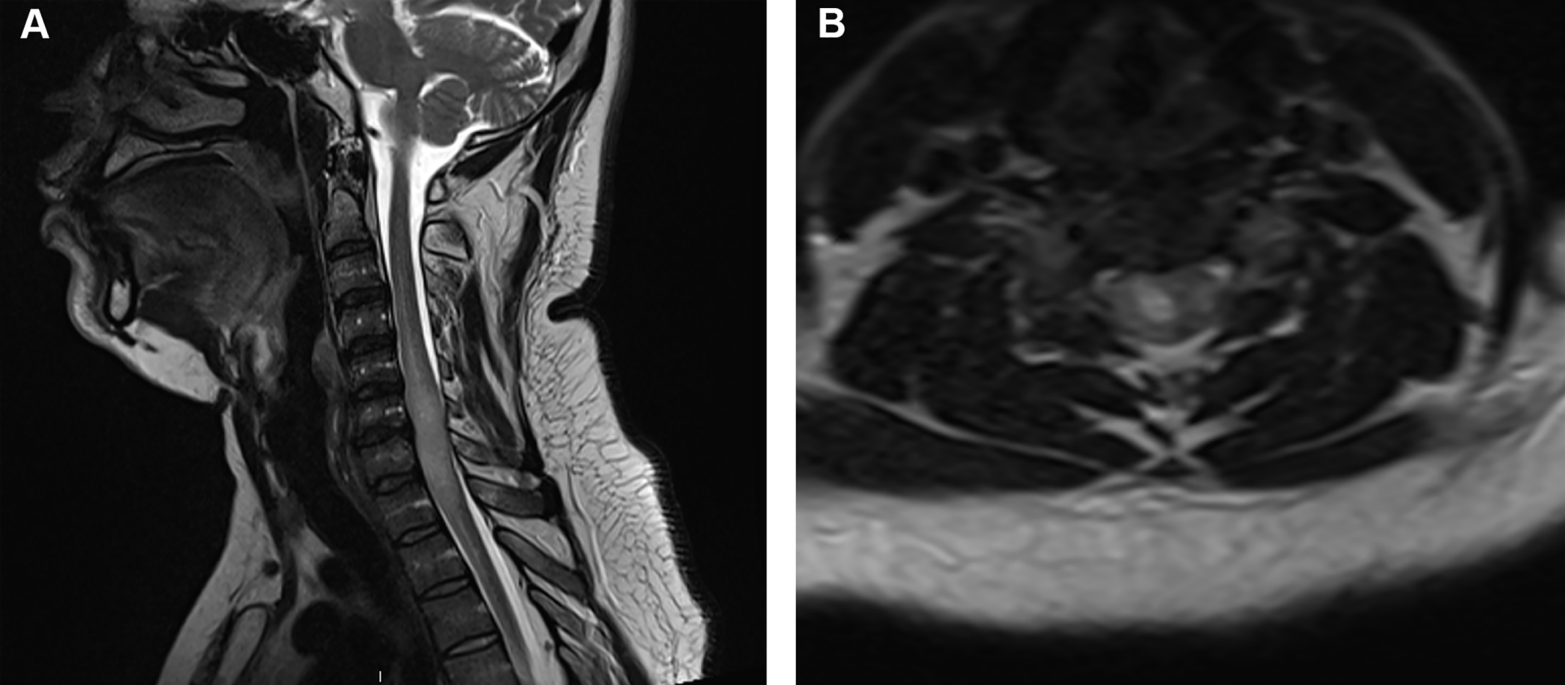
Adult patient with an intramedullary astrocytoma seen on **(A)** sagittal T2-weighted MRI and **(B)** axial T2-weighed MRI extending from the C4 to T3 vertebral levels with effacement of the subarachnoid cerebrospinal fluid. The patient presented with lower limb paralysis and loss of bladder and bowel function. Intra-operatively, a subtotal resection was performed with removal of visible tumor; however, the tumor was noted to be infiltrative. Pathology revealed an *H3K27M-*altered diffuse midline glioma resulting in a WHO Grade 4 diagnosis, although the histological appearance resembled anaplastic astrocytoma.

Surgical intervention is considered the mainstay of treatment in patients with intramedullary astrocytomas experiencing neurological deficits or myelopathy, with a less clear role for patients with pain or incidental findings. Although GTR is believed to improve local tumor control, it often cannot be achieved without risking significant post-operative morbidity to patients, given the infiltrative nature of astrocytomas and lack of clear margins (24–26). Consequently, surgeons must balance the potential improvement in survival from more extensive resections with the increased risk of worsening and/or new neurological deficits (27). Reported rates of GTR are lower compared to intramedullary ependymomas, which often present with distinct cleavage planes (17, 28, 29). Patients with low-grade astrocytomas, particularly pilocytic astrocytomas, may present with dissection planes that facilitate gross total resection; however, GTR can be difficult to achieve in high-grade astrocytomas (17, 28, 30). Using intra-operative findings and neuromonitoring, and considering the patient’s pre-operative baseline deficits, surgeons must determine the maximal safe resection feasible. Tumors lacking clear planes or presenting with imaging findings suggestive of a high-grade lesion should be considered for near total or subtotal resection (STR) with biopsy (**Figure 2**) (31). Patients with significant comorbidities, advanced age, or paraplegia should also be considered for STR when the benefits of GTR are expected to be minimal.

Patients are usually positioned in the prone position and a posterior midline approach used for subperiosteal dissection and removal of the bony elements relevant for the dural exposure. Mean arterial pressure should be preserved above 80–90 mmHg to maintain spinal cord perfusion (for pediatric patients, we advocate maintaining the pre-operative MAP values to slightly above it). The dura is opened in the midline and can be tacked to surrounding muscle tissue using dural tacking sutures (32, 33). Intra-operative ultrasound can be used to visualize the lesion and confirm location prior to myelotomy, minimizing cord manipulation. Intramedullary tumors generally appear hyperechoic on ultrasound compared to healthy spinal cord, and infiltrative astrocytomas may have blurry margins (34).

Thereafter, an operating microscope should be introduced to the field to minimize damage to cord tissue and identify dissection planes. A midline myelotomy between the dorsal columns is usually performed. The dorsal medial vein can be identified to help identify the midline. Other relative safe entry zones include the dorsal intermediate or dorsolateral sulci, also known as the dorsal root safe entry zone. For lesions positioned laterally, the midline myelotomy can be used, but alternative routes include the dorsal root entry zone or an anterior to dorsal root entry zone myelotomy can be performed (32, 35). Some advocate that the pia mater be tacked to the dura to maintain the operative field, although we prefer using dynamic retraction to minimize stress and tension on the spinal cord (36, 37).

Once the tumor is reached, fine dissection is made along the borders and disconnection of the tumoral blood supply is performed when feasible. In many cases, the main technique will include debulking of the tumor inner core followed by disconnecting and dissecting the tumor from the healthy spinal cord. This is carried out interchangeably, which again allows for dynamic retraction and tension on the spinal cord. An ultrasonic aspirator can be used to remove the tumor (38). Highly infiltrative astrocytomas should be considered for biopsy only, while tumors appearing low grade or those with a relatively clear plane can be attempted for complete removal (39). After hemostasis is achieved, a watertight dural closure can be performed, followed by closure of the superficial layers (39). Again, intra-operative ultrasound can be used to confirm the resection extent. An expansile duraplasty is sometimes performed after biopsy or debulking to provide increased space to reduce the risk of cerebrospinal fluid leak and alleviate clinical deficits resulting from tumor growth (36, 40, 41).

Intra-operative neuromonitoring should be performed to facilitate maximal safe resection. Studies have suggested improved functional outcomes for patients with intra-operative monitoring compared to those without monitoring (37, 42). Institutional studies have reported using somatosensory evoked potentials, motor evoked potentials, and transcranial motor evoked potential monitoring (17, 39). Loss of evoked potentials without recovery is often considered an endpoint for resection due to its ability to predict new post-operative deficits, although protocols differ (17, 32, 43). A systematic review suggested that motor evoked potentials have a sensitivity of 84% and sensitivity of 83% for detecting injury (44). Neuromonitoring with somatosensory and motor evoked potentials can involve a slight delay of several seconds from the time of insult to recognition. D-wave epidural or intradural monitoring is used by some surgeons due to its faster, real-time feedback, with a decrease in amplitude ≥50% often used as an endpoint (39, 43). D-waves are not influenced by blood pressure, heart rate, and anesthetic drugs, unlike motor and somatosensory evoked potentials. A review of 28 patients with intramedullary tumors illustrated that D-wave specificity, compared to motor and somatosensory evoked potentials, was most consistent over time, with a specificity nearly twice as large as motor evoked potentials (45). However, D-waves cannot always be obtained, particularly in patients with pre-existing deficits and patients with tumors located near the conus medullaris due to the limited tracts available (17, 43).

Both laminectomy and laminoplasty have been described for intramedullary surgery. Some studies have suggested that laminoplasty can reduce the likelihood of post-operative spinal deformity, particularly in children at risk of kyphotic deformity (46). Ahmed et al. noted that laminoplasty can help promote wound healing, reduce the risk of a CSF leak, and facilitate a safer extradural dissection plane in patients undergoing repeat surgery (47). However, a separate study of 66 pediatric patients undergoing resection of intramedullary tumors did not observe laminoplasty to result in a significantly different rate in the incidence of instrumented fusion for spinal deformity in pediatric patients undergoing resection of intramedullary tumors compared to those receiving laminectomy (48). Some surgeons prefer laminoplasty in pediatric cohorts; however, additional studies are needed (17). A spinous process-splitting hemi-laminoplasty could also be considered, although reports are lacking in the literature for intramedullary tumors (49).

Consensus is lacking concerning the optimal surgical treatment strategy and the effect of resection extent on post-operative outcomes (41). Several studies have failed to observe a significant effect of extent of resection in patients with intramedullary astrocytomas, and some have shown surgery to worsen functional outcomes (6, 21, 50). In contrast, others have identified a role for extent of resection independent of underlying histology for improving post-operative survival (24, 51). A national database study of 348 patients by Luksik et al. determined that GTR or partial resection significantly improve survival in children with intramedullary astrocytomas compared to those receiving biopsy only or no surgery (3). A separate national database study by Adams et al. of 135 patients with primary malignant intramedullary astrocytomas also established extent of resection as a significant predictor of survival (51). Resection can improve local control, delaying tumor progression and improving survival (52). A single-institutional study of 94 patients found that GTR was achieved in 13% of patients and a near-total resection achieved in another 34%. Their 10-year survival rate was 55%, although patients who survived 10 years largely had WHO Grades 1 and 2 tumors, as higher-grade tumor patients had shorter overall survival (17). Higher tumor grades were also significantly associated with shorter time to tumor progression after surgery, and chemotherapy did not improve survival (24). Separately, a French multi-center study of 95 patients achieved a GTR rate of 38% with 10-year survival of 77% (53). A systematic review by Hamilton et al. suggested that GTR improves overall survival in patients with intramedullary astrocytomas irrespective of underlying grade (54). However, this may come at the expense of worsening neurological deficits (41). Similarly, Golpayegani et al. conducted a meta-analysis of 1,079 patients and found that GTR significantly improved survival compared to STR on Kaplan–Meier survival analysis (31). In contrast, some authors advocate a more cautious approach, noting that GTR should be attempted where feasible, particularly in low-grade lesions, but cautioning against its use in high-grade lesions (41, 55).

New advances in intra-operative neuronavigation and imaging have been described for intramedullary tumors that may help facilitate a maximal safe resection. In addition to intra-operative B-mode ultrasound for tumor visualization, contrast-enhanced ultrasound has been reported to improve lesion localization and myelotomy planning with good correlation to pre-operative MRI. The contrast consists of microbubbles that can illustrate the tumor vascular network, and outline the tumor to a greater extent than B-mode imaging. In addition, contrast administration after resection can help confirm the resection extent (56). Augmented reality operating microscopes have been reported to help visualize tumor outline and assist with resection using principles similar to intracranial augmented reality-based applications (57). Similarly, multimodal neuronavigation techniques are limited but have been described, including one that incorporates diffusion tensor imaging, three-dimensional virtual reality imaging, and the operating microscope to visualize tractography in real time (58). Although these technologies are associated with learning curves, we anticipate that continued refinement of these technologies will lead to increased uptake by surgeons and help improve intramedullary lesion resection. In addition, we expect that improvements in tractography and diffusion tensor imaging will allow for improved pre-operative surgical planning to minimize the risks of neurological deficits (59, 60).

### Adjuvant therapy

Radiation therapy (RT) and chemotherapy are sometimes used in patients with high-grade lesions or patients with progressive disease following resection, with a less clear role following STR of low-grade lesions (61). Potential benefits in survival from RT must be balanced against the cord’s limited capacity to tolerate radiation and potential for injury (21). Additionally, RT in children can affect growth and result in radionecrosis and vasculopathy (62). The literature is conflicting on the therapeutic benefits of RT, and the optimal timing and dose of radiation are unclear (54). Some studies have not found any significant effect of RT on overall survival (6, 63). Other studies have suggested a deleterious effect on overall survival, although this may reflect selection bias as high-grade lesions with poor prognosis are more likely to receive RT (3, 24). A meta-analysis suggested that RT worsened outcomes for low-grade lesions but improved overall survival in high-grade astrocytomas (54). Similarly, MInehan et al. identified a survival benefit for infiltrative tumors but not pilocytic astrocytomas (64). RT is, therefore, often administered in patients with high-grade infiltrative lesions with a poor prognosis. Proton therapy has a sharp dose fall-off and can also be administered for spinal cord tumors, which may reduce damage to surrounding tissues and improve safety. However, like conventional RT, no clear benefit has been identified in the literature. Indelicato et al. found that intramedullary tumors treated with proton beam therapy had inferior local control compared to cerebellar and cerebral tumors, whereas Kahn et al. found on multivariable analysis that 10 patients with intramedullary tumors treated with proton beam therapy fared worse than those treated with photon beam RT (65, 66). The authors suggest that the sharp dose fall-off may limit effective treatment for highly infiltrative tumors (66).

Similarly, the efficacy of chemotherapy is unclear, with limited data supporting its use (67). It is usually reserved for high-grade lesions that recur or progress despite resection or RT, or for pediatric patients less than 3 years old in whom RT is contraindicated (17, 21, 24, 68). Some case series have reported promising results using chemotherapy for intramedullary astrocytomas, favoring its use for recurrent or unresectable lesions (21, 62, 69). However, a systematic review of nine studies of patients with spinal cord glioblastoma treated with temozolomide did not confer a significant survival benefit, although owing to the rarity of the disease, the sample size only included 19 patients (70). Chemotherapeutic regimens are heterogenous, although temozolomide is often administered based on the Stupp protocol employed for intracranial GBM (24, 71). Other agents include carboplatin, vincristine, vinblastine, and irinotecan (24).

Novel treatment strategies are necessary to improve outcomes in patients with spinal cord astrocytomas, particularly those with high-grade aggressive lesions not amenable to GTR. Outcomes remain poor in these patients, with average overall survival of only a few months (3, 17, 21). Furthermore, although the WHO grade is an important prognosticator, not all patients with the same histological grade will experience the same outcomes. Lesions of the same grade can differ in their degree of infiltration and prognosis, suggesting that important genetic and molecular differences exist that can inform the optimal treatment paradigm. Moreover, identification of genetic mutations and molecular biomarkers may represent an opportunity for personalized, targeted therapy, offering patients a new option for treatment beyond surgical resection.

## Molecular landscape

The past decade has seen increasing interest in identifying the genomic landscape of intramedullary astrocytomas. Detection of target genes or biomarkers can improve prognostication and may represent novel targets for personalized or genetic therapy. Molecular markers were included in the WHO classification of central nervous system tumors for the first time in 2016, which defined new lesions and subcategorized tumors based on genetics. For example, the guidelines defined the entity known as diffuse midline glioma H3 K27M-mutant to refer to midline tumors primarily found in children with K27M mutations in the histone H3 gene H3F3A (72). Glioblastomas were also categorized based on IDH mutation status.

Most recently, the 2021 WHO classification substantially updated the classification of tumors with a greater emphasis on genetic, epigenetic, and molecular markers in addition to traditional histological grading. IDH-mutant astrocytomas are considered a distinct entity and categorized as Grade 2, 3, or 4, while CDKN2A/B deletions provide further subclassification to IDH-mutant astrocytomas (7). In some cases, the presence of a particular genetic mutation is sufficient to determine the tumor grade irrespective of the underlying histological appearance. Glioblastoma can be defined as an IDH wild-type, H3 wild-type tumor with *TERT* promoter mutation, *EGFR* amplification, +7/-10 chromosome copy-number alterations, microvascular proliferation, or necrosis, illustrating the aggressive nature of IDH wild-type tumors that were previously categorized only as Grade 2 or 3 based on histological appearance (73). In addition to recognition of genetic mutations, alterations of molecular pathways, such as the MAPK pathway, are also considered for tumor categorization (7).

Importantly, the increase in knowledge of astrocytoma genetics has mainly been focused on intracranial astrocytomas and may not necessarily translate to spinal cord astrocytomas. Like we see with the appearing anatomical distinction in ependymomas (stating supratentorial, infratentorial, and spinal cord ependymomas are different tumors), there is high likelihood that in the future, we will learn distinctive features between spinal cord astrocytomas and the intracranial ones.

Although the genetics of intracranial astrocytomas have been extensively studied, intramedullary astrocytomas have been shown to harbor a unique set of genetic mutations and molecular biomarkers (**Table 1**) (82–84). Therefore, chemotherapeutic and targeted therapies designed for intracranial astrocytomas may not translate successfully to spinal cord neoplasms, whose underlying genetic mutations inform their natural history and response to therapy (83). As an example, Bettegowda et al. have noted that the frequency of *KIAA1549-BRAF* fusions in spinal pilocytic astrocytomas is lower than the reported frequency in their intracranial counterpart (85). Moreover, Lebrun et al. illustrated that the breakpoints involved in the *KIAA1549-BRAF* fusion differed in their frequency between intracranial and intramedullary pilocytic astrocytomas (86). Consequently, a thorough understanding of the molecular landscape of intramedullary tumors is critical for improving treatment strategies and aiding surgical management.

**Table 1:** Genetic mutations frequently associated with intramedullary astrocytomas.

Gene	Locus	Mutation	Function	Notes	Targeted therapy
*H3F3A*	1q42	Missense	Histone protein; mutation alters epigenetic regulation of expression of oncogenes	Poor prognosis, classified as WHO Grade 4	Inhibition of demethylases to increase histone methylation, EZH2 inhibitors to prevent heterochromatin formation, CAR T-cell immunotherapy (74).
*MGMT*	10q26	Methylation of promoter	Removes methyl groups from guanine, countering alkylating agents; mutation renders tumors more susceptible to alkylation	Improved prognosis in high-grade astrocytomas	MGMT inhibitors e.g. O^6^-benzylguanine, O^6^-(4-bromothenyl) guanine (75).
*BRAF*	7q34	Fusion with KIAA1549 or missense	Regulates cell growth, proliferation, differentiation, tumorigenesis; mutation results in constitutive activation	Improved prognosis, frequent occurrence in pilocytic astrocytomas	BRAF-MEK inhibitors, e.g., vemurafenib/cobimetinib (76), dabrafenib/trametinib, encorafenib/binimetinib (77).
*IDH1*	2q34	Missense	Citric acid cycle enzyme, mutation produces R-2-hydroxyglutarate that alters epigenetic regulation	Improved prognosis, but low frequency in intramedullary astrocytomas	IDH1 inhibitor ivosidenib (78).
*TP53*	17p13	Missense	Cell cycle regulation, tumor suppressor; mutation promotes tumor formation and growth	Associated with secondary GBM, linked to Li-Fraumeni syndrome	Suppression of mutant p53, restoring wild-type conformation and activity (79).
*CKN2A*	9p21	Deletion	Tumor suppressor proteins that induce cell cycle arrest; mutation drives proliferation	Poor prognosis	
*CDK4*	12q14	Amplification	Promotes cell cycle progression; mutation drives proliferation	Poor prognosis	CDK4 inhibitor Palbociclib (80).
*ATRX*	Xq21	Missense, deletion, fusion	Chromatin remodeling and epigenetic regulation; mutation alters genetic expression	Associated with *IDH1* mutations, more common in high-grade intramedullary astrocytomas than low-grade	Restoring native chromatin configuration, DNA-damaging agents (81).

CAR, chimeric antigen receptor; WHO, World Health Organization.

### H3K27M

Somatic missense mutations at amino acid position 27 of the *H3F3A* or *HIST1H3B* genes are commonly seen in astrocytomas of midline structures, including the pons, thalamus, and spinal cord, resulting in the discrete entity designated diffuse midline glioma, H3 K27M-altered (87). They are estimated to account approximately between 18% and 55% of intramedullary astrocytomas (86, 88, 89). Originally designed in the 2016 WHO guidelines as H3 K27M-mutant lesions, the updated nomenclature reflects other mechanisms can alter the pathway beyond genetic mutations (7). Most commonly, a missense mutation change a lysine to methionine residue (K27M) in the histone H3 variants H3.3 or H3.1, resulting in a change in genetic expression that is believed to drive tumor formation and growth (87, 90). These gliomas are also associated with a loss of trimethylation in residual H3K27 and an increase in H3K27 acetylation, thereby upregulating expression of proto-oncogenes and suppressing cellular differentiation (90, 91).

H3 K27M-altered diffuse midline gliomas are aggressive lesions most commonly appearing in children and young adults (**Figure 2**) (87, 92). Although they can appear in several midline structures, the spinal cord is the most common site of H3 K27M-altered gliomas in adults (93). They are designated WHO Grade 4, although their underlying microscopic appearance may appear Grade 2, 3, or 4 (88, 94). Indeed, the majority of H3 K27M-altered gliomas appear as high-grade lesions, but approximately 40% can appear as Grade 2 in histology (88, 95). Cheng et al. studied 59 patients with intramedullary astrocytomas, of which 28 were designated H3K27M-altered, and found that shorter symptom duration was significantly associated with the H3K27M mutation, but did not identify significant differences on MRI (88). Gu et al. reported five cases of intramedullary diffuse midline gliomas with the H3K27M-alteration and noted that all were initially mistaken as benign based on their radiographic appearance (93). Interestingly, the H3K27M-alteration has not been shown to present alongside *IDH* mutations, suggesting the two are mutually exclusive (96, 97).

H3 K27M-altered tumors portend a poor prognosis and significantly worsen overall survival for histological Grade 2 lesions, although histological Grade 2 lesions bearing the H3 K27M-alteration have improved survival compared to histological Grade 4 H3 K27M-altered gliomas (95). Several studies have shown that they do not significantly worsen survival in histologic Grade 3 or 4 lesions compared to high-grade wild-type astrocytomas, illustrating that histological grade is still an important consideration in evaluating these lesions, although statistical significance in those studies may have been limited by small sample sizes (88, 95). Adults with the H3K27M-altered gliomas may have improved prognosis compared to pediatric patients (93). Inhibition of the histone demethylase JMJD3 can increase H3K27 methylation and has shown encouraging results in *in vitro* and *in vivo* mice models of brainstem glioma, illustrating the importance of genetic analysis of astrocytomas to identify potential targeted therapeutics and improve patient outcomes (98). The investigational compounds imipridone ONC201 and IDO1 inhibitor indoximod are also being investigated for treatment of H3K27M-altered gliomas both intracranially and in the spinal cord (74, 99).

### MGMT

The O6-methylguanine-DNA methyltransferase (MGMT) protein is a DNA repair enzyme that repairs damaged guanine nucleotides by transferring methyl groups from the guanine O^6^ site to cysteine residues, thereby providing resistance to alkylating agents such as temozolomide (TMZ) that methylate purine bases (100, 101). Epigenetic methylation of CpG islands of the *MGMT* promoter results in heterochromatinization and downregulation of the gene, increasing susceptibility of tumors to alkylating agents (75). Consequently, several clinical trials have illustrated that *MGMT* promoter methylation is associated with improved survival compared to unmethylated tumors and is an independent prognostic factor in high-grade astrocytoma (102, 103). Indeed, a meta-analysis of 34 studies by Binabaj et al. illustrated that *MGMT* methylation improves overall survival in GBM patients compared to unmethylated patients (104). Interestingly, *MGMT* promoter methylation is rarely observed in H3K27M-altered gliomas. Consequently, the increased preponderance of the H3K27M-alteration in intramedullary astrocytomas compared to intracranial astrocytomas may explain the former’s decreased responsiveness to temozolomide (105).

Stratification of intramedullary astrocytomas by *MGMT* methylation status can help assess prognosis and determine the value of adjuvant chemotherapy compared to resection and radiotherapy. Rovin and Winn described a 28-year-old woman with a high-grade intramedullary astrocytoma lacking expression of *MGMT* whose residual tumor regressed favorably after surgery, radiation, and temozolomide, illustrating the significance of *MGMT* methylation status (106). Sun et al. classified 47 spinal cord gliomas based on *MGMT* expression, administering TMZ alone to patients with negative expression but providing both TMZ and the anti-neoplastic agent cisplatin to patients with positive expression (100). Other targeted therapies are being studied, including MGMT inhibitors such as O^6^-benzylguanine and O^6^-(4-bromothenyl) guanine (75).

### BRAF

Genetic changes in the *BRAF* gene are frequently found in pilocytic astrocytomas, a low-grade glioma common in the pediatric population (107, 108). *BRAF* encodes the B-raf protein, an intracellular serine/threonine kinase involved in the mitogen-activated protein kinase pathway (MAPK) regulating cellular growth, proliferation, differentiation, and tumorigenesis (107). Fusion between *BRAF* and *KIAA1549* results in loss of the *BRAF* N-terminal auto-inhibitory domain, producing a fusion oncogene and constitutive activation of the MAPK pathway (109, 110). The *BRAFV600E* point mutation has also been described in pilocytic astrocytomas, as it disrupts the auto-inhibitory mechanism of B-raf, thereby promoting B-raf activation (109). *BRAF* fusions are associated with a favorable prognosis, in part related to B-raf’s role in promoting oncogene-induced senescence in tumors, as well as its frequent occurrence in pilocytic astrocytomas, which inherently have favorable outcomes (109, 111–113).

Although *BRAF* changes can occasionally be found in higher grade gliomas, they mostly occur in pilocytic astrocytomas. The *KIAA1549-BRAF* fusion is estimated in nearly 80% of cerebellar pilocytic astrocytomas, but only around 50% of non-cerebellar pilocytic astrocytomas, including those of the spinal cord (86). Chai et al. observed the *BRAF V600E* mutation in 12% of intramedullary astrocytomas, noting its occurrence only in H3K27M–wild-type neoplasms (95). They also noted significantly improved survival for histological Grade 2 or 3 astrocytomas with a *BRAF V600E* mutation compared to Grade 2 or 3 wild-type astrocytomas (95). Of note, the frequency of fusion breakpoint location differ between intracranial and intramedullary astrocytomas harboring a *KIAA1549-BRAF* fusion. Lebrun et al. noted that the *KIAA1549 (* 15)*-BRAF(9)* fusion was most common in intramedullary pilocytic astrocytomas, identified in 80% of tumors with the fusion oncogene, while the *KIAA1549(16)-BRAF(9)* fusion was most common in intracranial pilocytic astrocytomas (86). The implications are not entirely elucidated, but nonetheless illustrate the distinct molecular landscape of intramedullary astrocytomas compared to their intracranial counterparts. BRAF-MEK inhibitors can potentially be used to improve outcomes in patients harboring *BRAF* fusions or mutations (84, 114). Balasubramanian et al. treated an adult patient with a *BRAF V600E* pilocytic astrocytoma with vemurafenib, a B-raf inhibitor, and cobimetinib, a MEK inhibitor, with 1-year follow-up showing tumor stability and reduction of T2 cord signal cranial and caudal to the lesion (76).

### IDH

Mutations in the isocitrate dehydrogenase (*IDH*) gene are common in intracranial Grades 2 and 3 astrocytomas; however, their prevalence is rarer in intramedullary astrocytomas (115). IDH1 and IDH2 are enzymes responsible for conversion of isocitrate in the citric acid cycle, and mutations result in accumulation of R-2-hydroxyglutarate (116). This metabolite competitively inhibits histone demethylases, resulting in broad epigenetic changes to DNA methylation and gene expression (83). *IDH* mutations can be used intracranially as both a diagnostic marker to distinguish diffuse from pilocytic astrocytomas, given its rare incidence in pilocytic astrocytomas, and as a prognostic marker, with IDH-mutant tumors having a more favorable prognosis compared to wild-type tumors (115, 117).

The prognosis of *IDH* mutations is less clear for intramedullary astrocytomas, given their low frequency (84, 85). Indeed, a review of 58 patients with spinal cord astrocytomas by Chai et al. did not identify any *IDH* mutations (95). Five cases of *IDH1* mutations in intramedullary astrocytomas were noted by Konovalov et al. using next generation sequencing, noting that rare and unique variants were identified that are not conventionally tested using immunohistochemistry. Therefore, the true prevalence of *IDH1* mutations in spinal cord astrocytomas may be greater than reported, as only the dominant intracranial R132H variant is generally tested using immunohistochemistry (115). Similarly, Takai et al. reported an *IDH1* mutation in an adult female with a histological Grade 2 intramedullary diffuse astrocytoma. A R132S mutation was noted using Sanger sequencing, again differing from the dominant R132H traditionally examined (118). Although *IDH* mutations are generally a favorable prognostic factor, the patient only survived 11 months, below the average expected for astrocytomas of similar histological grade. In contrast, Nagashima et al. reported R132C and R132H mutations in two patients with Grade 2 intramedullary astrocytomas, noting a favorable prognosis with both patients alive with 22–37 months of follow-up (119). Next generation sequencing and identification of larger cohorts of *IDH*-mutant intramedullary astrocytomas is needed to determine its prognostic value.

### TP53

The tumor suppressor protein p53 is encoded by the *TP53* gene and is involved in regulation of the cell cycle, including cell cycle arrest and apoptosis upon detection of damage (120). *TP53* is altered in over 20% of GBMs, but is also commonly mutated in lower grade adult diffuse astrocytomas (86, 121, 122). Mutation promotes initiation, maintenance, and progression of tumor growth and has been linked to adverse outcomes (123). Lebrun et al. found that *TP53* mutations were present in 21% of examined intramedullary astrocytomas, including 53% of high-grade lesions. In particular, H3K27M-mutant astrocytomas had the highest rate of *TP53* mutations, accounting for 64% of cases (86). The median survival of five patients with intramedullary *TP53*-mutated astrocytomas was reported by Alvi et al. as 11.5 months (124). Noor et al. suggested that outcomes after chemotherapy may be improved in low-grade gliomas with a *TP53* mutation; however, this study was not specific to intramedullary astrocytomas (120).

Deregulation of *TP53* can promote overexpression of mutant-p53, which accumulates in the nucleus and drives tumorigenesis through its interaction with oncoproteins (120, 121). Under physiological conditions, p53 expression levels are kept low unless activated by cellular stress or DNA damage (125). Mutant-p53 has a longer half-life compared to wild-type–p53 and can, thus, be more readily detected by immunohistochemistry (125). Both *TP53* mutations and overexpression of p53 correlate with a poor prognosis (122). Similar to *TP53* mutations, p53 overexpression is particularly found in high-grade astrocytomas. Govindan et al. detected immunoreactivity for p53 in 5/6 intramedullary GBMs, while Santi et al. reported p53 in 7/10 intramedullary high-grade astrocytomas (126, 127). High levels have been reported especially in secondary GBM, which are tumors that developed from lower grade lesions (127). Of note, Sarkar et al. reported high p53 expression in astrocytomas that progressed to GBM compared with recurrent tumors that remained at the same histological grade, suggesting inactivation of the p53 tumor suppressor pathway may play a role in tumor progression to GBM (127, 128).

### Cyclin-dependent kinases

Cyclin-dependent kinases play a critical role in cell cycle regulation. Homozygous deletion of the *CDKN2A* locus is associated with a poor prognosis and is associated with progression to high-grade gliomas (129). The locus encodes the p14 and p16 tumor suppressor proteins, which induce cell cycle arrest, such that deletion of the gene promotes cellular proliferation (130). Although frequently encountered in astrocytomas, few studies have examined *CDKN2A* deletion specifically in intramedullary tumors, although Horbinski et al. noted that homozygous deletion was seen more frequently in tumors of the spinal cord, midbrain, and brainstem than those of the cerebellum or cerebrum (131). These deletions have particularly been found in *IDH*-mutant astrocytomas, leading some to argue that the *IDH*-mutant category should be stratified further using genetic markers such as *CKDN2A* (132, 133). Lu et al. conducted a systematic review of nine studies analyzing a total of 2,193 *IDH*-mutant gliomas, concluding that homozygous deletion of *CDKN2A* was a significant predictor of both shorter progression-free and overall survival in astrocytomas. However, the study was not specific to spinal cord astrocytomas, and further research is needed in this population.

Relatedly, *CDK4* amplification has been associated with poor outcomes in astrocytomas (133). Unlike p14 and p16, CDK4 drives cell cycle progression by forming a complex, which releases the transcription factor E2F from the Rb protein (134). Therefore, both *CDK4* amplification and deletion of *CDKN2A* function to promote cellular proliferation. Lin et al. described a refractory intramedullary astrocytoma with *CDK4* amplification detected on next generation sequencing. The patient underwent two surgeries and received radiation therapy, TMZ, and bevacizumab, but nonetheless progressed. Treatment with Palbociclib, a selective *CKD4* inhibitor, was effective at promoting tumor regression, and the patient was alive at the 2-year follow-up visit, illustrating the potential of personalized therapy for patients with intramedullary tumors (80).

### ATRX

The *ATRX* gene regulates chromatin remodeling and genetic stability by depositing the histone variant H3.3 at heterochromatin and telomeres (83, 135). Inactivation can arise from mutations, deletions, and fusions, and is strongly associated with *IDH1* mutations in diffuse and anaplastic astrocytomas (83, 135). Although most studies have investigated their frequency and prognosis in intracranial lesions, Lebrun et al. confirmed an increased prevalence of *ATRX* mutations among high-grade intramedullary astrocytomas compared to low-grade astrocytomas, detecting mutations in 33% of histological Grade 3 and 4 tumors, but only 13% of Grade 1 and 2 tumors (86). Overall, 10/61 cases were reported with *ATRX* mutations. Additionally, they were noted to occur more frequently in H3K27M–wild-type intramedullary astrocytomas compared to H3K27M-mutant astrocytomas. Yuzawa et al. reported a case of a 27-year-old man with neurofibromatosis Type I with a histological Grade 2 tumor. *ATRX* immunoreactivity was retained; however, residual tumor progressed in size 7 years later and appeared under histology as GBM with loss of *ATRX* expression. The case suggests that loss or inactivation of *ATRX* can be associated with malignant transformation of astrocytoma (136). Tumors lacking *ATRX* expression can potentially be targeted with epigenetic strategies aimed at restoring proper chromatin configuration. In addition, these tumors may be sensitive to pharmaceutical agents that damage DNA, given the genomic instability that arises in *ATRX*-mutant cells (81).

## Discussion

Treatment strategies for primary intramedullary astrocytomas are centered around surgical resection of symptomatic lesions causing neurological deficits. The fact that treatment still relies mainly on the results of surgical resection reflects the lack of meaningful results from adjuvant therapy, as well as the lack of knowledge about the genetic features of intramedullary astrocytomas that would allow for targeted therapy. Although GTR or near-total resection can be achieved for well-circumscribed tumors presenting with a distinct cleavage plane, most astrocytomas infiltrate the cord and lack clear margins, precluding GTR without worsening the patient’s functional status and neurological deficits (137). Consequently, rates of GTR are low for intramedullary astrocytomas, while STR and biopsy are often the only feasible choices (24). For example, one retrospective study of 94 patients with intramedullary astrocytomas established a GTR rate of 13%, near-total resection rate of 34%, STR rate of 44%, and biopsy rate of 10% (24). Similarly, another institutional study of 12 patients with astrocytomas found that a dissection plane could be developed in only 29% of cases, compared to nearly all ependymomas (138). Nonetheless, the maximal safe resection can often be a near-total resection, and extent of resection has been associated with overall survival (24). Adjuvant radiation and chemotherapy can be provided, but their efficacy is not well-established, with conflicting reports in the literature on their impact on survival (24). Overall survival remains particularly poor in high-grade intramedullary astrocytomas, with a 5-year overall survival rate of approximately 14% for histological Grade 4 astrocytomas (17, 139). However, most studies investigating survival outcomes and the efficacy of resection have focused on histological grading systems from prior WHO categorization systems, rather than the updated 2021 guidelines incorporating molecular features.

Identification of molecular markers in tumor samples may help improve detection, assessing prognosis, and treatment of intramedullary tumors. Tumors with a clear plane can still undergo GTR during the index surgery, as the molecular features will not be known until the tumor specimen is analyzed post-operatively. However, STR and biopsy may suffice for most astrocytomas when the goal of surgery is obtaining sufficient tissue to identify genetic alterations. The tumor can be examined for its responsiveness to RT, TMZ chemotherapy, and targeted therapy, thus reducing the occurrence of post-operative neurological deficits associated with maximal resection of infiltrative lesions. In cases with a large tumor burden where the adjuvant treatment is not known to be beneficial, increasing the resection extent may be advocated before initiating adjuvant treatment.

Advances in liquid biopsy technology may replace the need for an invasive surgical biopsy. Short fragments of circulating tumor DNA can be detected in cerebrospinal fluid and peripheral blood, offering a noninvasive method of assessing tumor mutational burden (140). Several studies have illustrated the feasibility of detecting DNA from H3K27M-mutated diffuse midline gliomas in cerebrospinal fluid (140). Methodologies to rapidly sequence *IDH, TP53*, and *ATRX* mutations in cerebrospinal fluid have also been described (141). However, this work has mainly been performed for intracranial tumors, and clinical validation is needed in intramedullary cohorts, where anatomic sequestration may limit its role (142). Translation of the technology to intramedullary tumors may allow for sampling of tumor DNA prior to any surgical intervention, providing the surgeon, oncology team, and patients with a set of defined mutations informing them of prognosis and treatment options. The benefit would be greatest in high-grade tumors with infiltrative margins where GTR is not feasible and surgical intervention carries substantial risks. Patients with high-grade lesions deemed resistant to radiation, chemotherapy, and targeted therapy can decide on the benefits of surgical debulking. Liquid biopsies can also allow for monitoring of tumors throughout treatment, offering an enhanced view of tumor evolution and regression and the effectiveness of targeted therapy (143).

The effectiveness of conventional radiation and chemotherapy for intramedullary astrocytomas is limited (24). However, by defining the key mutations in a tumor, either through surgical or liquid biopsy, an opportunity exists for personalized and targeted therapy. Although much of the research on targeted therapies is focused on intracranial tumors, treatment may be extended to astrocytomas of the spinal cord harboring the same genetic mutations. Further work is needed to confirm findings restricted to intracranial cohorts, given that subtle genetic differences may account for discrepant responses. Dabrafenib, a B-raf inhibitor, and trametinib, a MEK inhibitor, can be administered to patients with overactivation of the MAPK pathway stemming from a *BRAF V600E* mutation. These drugs are administered for melanomas and lung cancers harboring *BRAF V600E* mutations, and the combination showed meaningful responses in 33% of high-grade and 69% of low-grade gliomas in a Phase II clinical trial (144). Another trial of dabrafenib in pediatric patients with refractory or progressive *BRAF V600E* tumors illustrated an objective response rate in 44% of patients, larger than the response traditionally observed with chemotherapy, although the results may be limited to intracranial tumors (145). Additionally, several *IDH1* inhibitors are being tested for treatment of *IDH*-mutant astrocytomas, including ivosidenib and enasidenib. Identification of an *IDH* mutation also suggests that the tumor may have increased susceptibility to agents that damage DNA, along with conventional TMZ therapy, given the association between *IDH* mutations and impaired DNA repair (78). MGMT analogs, such as O^6^-benzylguanine and O^6^-(4-bromothenyl) guanine, can inactivate MGMT and sensitize gliomas to TMZ (75). A Phase II trial combining O^6^-benzylguanine with Gliadel wafers, or carmustine implants that deliver local chemotherapy, showed efficacy in patients with recurrent GBM, although patients were not stratified on *MGMT* status (146). The immune system can also be directed against molecular markers on astrocytomas by genetically modifying cytotoxic T lymphocytes to express a chimeric antigen receptor that recognizes a specific tumor antigen (147, 148). A Phase I clinical trial was reported showing that chimeric antigen receptor T cells can target the disialoganglioside GD2 expressed on *H3K27M*-mutant diffuse midline gliomas with clinical and radiographic improvement noted in three of four patients (149).

Challenges persist in the successful integration of targeted therapy with surgical treatment, RT, and chemotherapy for intramedullary astrocytomas. Despite an increasing number of reports in the literature concerning the mutational landscape of intramedullary astrocytomas, most studies have been limited to intracranial lesions. Important differences in the frequency and types of mutations have been noted across the two regions—for example, although *KIAA1549-BRAF* fusions are detected in astrocytomas in both regions, their frequency and breakpoint locations have been noted to differ (85, 86). The rarity of intramedullary astrocytomas has posed a challenge to accumulating large cohorts for genetic and epigenetic analysis. Consequently, research into targeted therapies is often limited to intracranial, rather than intramedullary, gliomas, although some trials, such as the imipridone ONC201 drug for H3 K27-M altered gliomas, include patients with spinal cord tumors (99). Additionally, biopsy specimens of spinal cord tumors are often small, given the tumors’ size and infiltrative nature, which can render genetic analysis difficult (83). Moreover, chemotherapy and targeted treatments can be limited by the blood-spinal cord barrier, which functions similarly to the blood-brain barrier to form a specialized micro-environment and restrict diffusion of large molecules into the central nervous system (150). Novel treatment strategies are being designed to improve permeability of the blood-spinal cord barrier, including the use of focused ultrasound to cause microbubble-induced transient opening of the barrier and improve drug delivery (151, 152).

Furthermore, although genetic differences exist between intracranial and intramedullary tumors, considerably less research has investigated differences across tumors by location in the spinal cord. It is reasonable to suspect that further sub-classification of tumors is possible within the spinal cord, just as genetic differences exist for intracranial astrocytomas according to region of the brain. Intramedullary tumors are usually reported with greater frequency in the cervical cord compared to the thoracic cord, which some authors’ suggestion reflects the higher density of gray matter in the cervical location (17, 153). Future investigations should examine whether cervical and thoracic astrocytomas differ in their genetic landscape.

**Figure 3:**
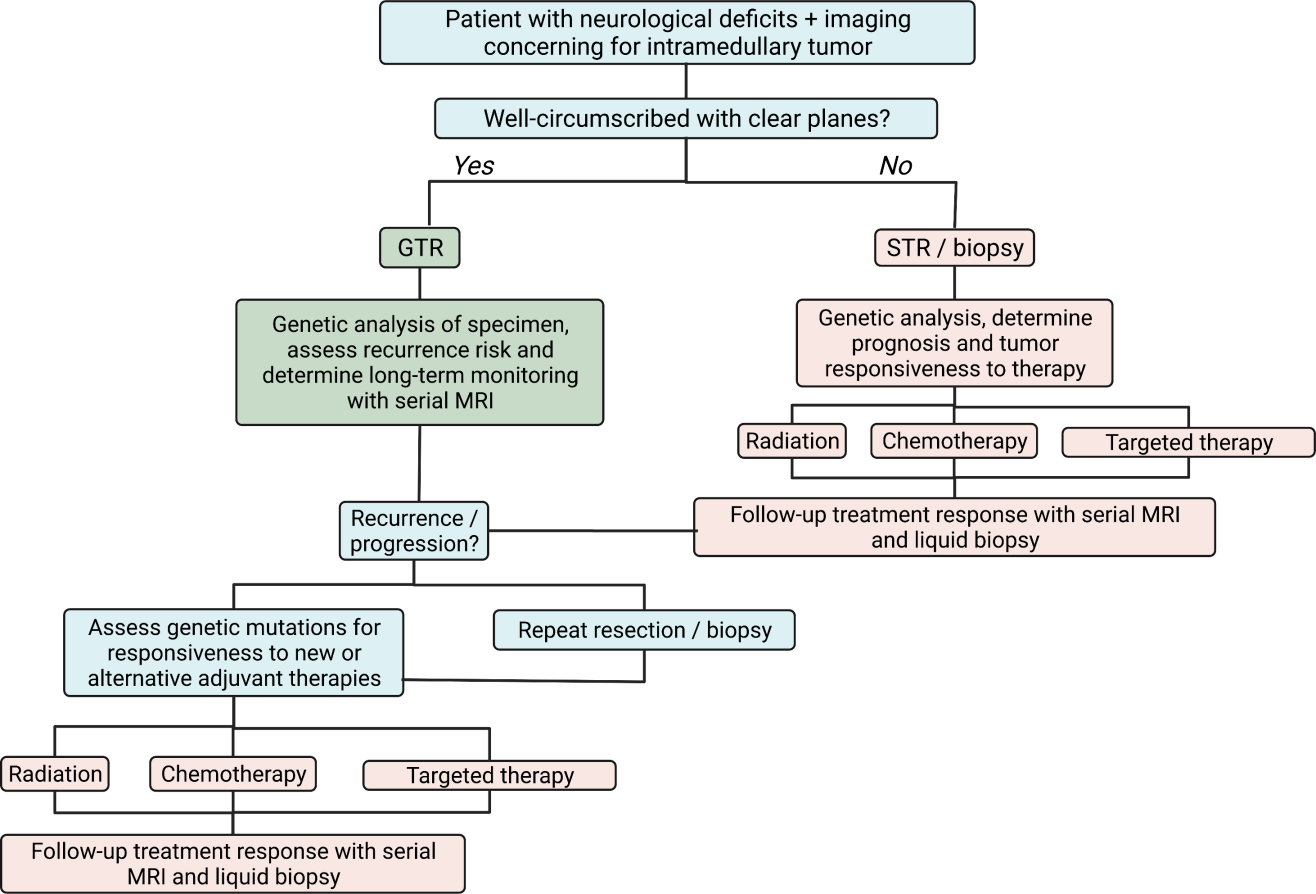
Algorithm to guide surgical decision-making and adjuvant treatment in patients with intramedullary astrocytomas using genetic analysis of tumors. Created with BioRender.com.

The dismal prognosis of intramedullary astrocytomas, particularly high-grade lesions, may eventually be improved by combining traditional surgical management with molecular analysis and targeted therapy. An algorithm that may guide future surgical management is presented in **Figure 3**. Symptomatic patients with well-circumscribed tumors and distinct cleavage planes, often seen in pilocytic astrocytomas, may undergo GTR with careful attention to neuromonitoring. In contrast, patients with diffuse, infiltrative astrocytomas may warrant only an STR with biopsy to identify the genomic features of the astrocytoma. Subsequent treatment can be tailored to the tumor using radiation, TMZ, and targeted agents, and liquid biopsies and serial MRIs can be used to monitor treatment response. A combination of agents may be needed, given the diverse range of mutations and heterogeneity seen across tumor cells within a patient. Recurrent or progressive tumors can be analyzed for new genetic markers that may represent opportunities for additional therapy. Tumors broadly resistant to targeted agents may be candidates for repeat resection after a discussion with the patient about their prognosis and surgical risks.

## Conclusion

Intramedullary astrocytomas are rare tumors that frequently present with infiltrative margins. Surgical resection using a midline myelotomy or dorsal root entry zone myelotomy is considered the mainstay of treatment; however, GTR is often not feasible in diffuse lesions without substantial risk of new post-operative deficits. Overall survival in high-grade astrocytomas is poor with few available treatment options. Molecular analysis of spinal cord tumors can help determine prognosis and therapeutic opportunities. Mutations in *H3K27M, MGMT, BRAF, IDH, TP53, CDKN2A, CDK4*, and *ATRX* have been described in these lesions and influence the aggressiveness and behavior of the tumor. Biopsy specimens from surgical resection can be analyzed to determine the unique genomic landscape of an intramedullary astrocytoma, and assess its responsiveness to radiation, chemotherapy, and targeted therapy, allowing surgical and oncological teams to select an optimal treatment regimen. Multi-institutional large cohorts are needed to validate the frequency and implications of genetic mutations in intramedullary astrocytomas, and clinical trials are of critical need to determine therapeutic agents for personalized therapy.

## Author contributions

Conceptualization – NS and GJ. Investigation - AH. Writing: Original Draft - AH. Writing: Review and Editing – NS and GJ. Visualization – AH and GJ. Supervision – NS and GJ. All authors contributed to the article and approved the submitted version.

## Conflict of interest

The authors declare that the research was conducted in the absence of any commercial or financial relationships that could be construed as a potential conflict of interest.

## Publisher’s note

All claims expressed in this article are solely those of the authors and do not necessarily represent those of their affiliated organizations, or those of the publisher, the editors and the reviewers. Any product that may be evaluated in this article, or claim that may be made by its manufacturer, is not guaranteed or endorsed by the publisher.

## Abbreviations

GBM, Glioblastoma; GTR, Gross total resection; IDH, Isocitrate dehydrogenase; MGMT, O6-methylguanine-DNA methyltransferase; MRI, Magnetic resonance imaging; RT, Radiation therapy; STR, Subtotal resection; TMZ, Temozolomide; WHO, World Health Organization.
